# Pseudogout as a Cause of Fever of Unknown Origin Following Staphylococcal Bacteremia in an Older Patient

**DOI:** 10.7759/cureus.24333

**Published:** 2022-04-21

**Authors:** Ryoko Yamauchi, Ryuichi Ohta, Mari Igarashi, Yasuo Kurita, Miho Hayakawa, Chiaki Sano

**Affiliations:** 1 Rehabilitation, Kanagawa Rehabilitation Hospital, Atugi, JPN; 2 Communiy Care, Unnan City Hospital, Unnan, Shimane, JPN; 3 Education, International University of Health and Welfare, Tokyo, JPN; 4 Cardiology, International University of Health and Welfare, Tokyo, JPN; 5 Medicine, International University of Health and Welfare, Tokyo, JPN; 6 Community Medicine Management, Shimane University Faculty of Medicine, Izumo, JPN

**Keywords:** japan, rural hospital, older patient, pseudogout, diagnosis, patients, fever

## Abstract

The causes of fevers in older adults are numerous and diverse, resulting in fevers of unknown origin that complicate the diagnosis process. Compared to young adults, older adults are characterized by comorbidities, aging-induced physiological changes, decreased homeostasis, reduced activities of daily living, and a diminished quality of life due to disease and aging. Thus, diverse perspectives are required to facilitate the accurate diagnosis of fever in older adults. In this study, we experienced a case of epidermal staphylococcal bacteremia of unknown cause with a persistent fever that eventually led to the diagnosis of cervical pseudogout. A 94-year-old bedridden woman visited our hospital with a chief complaint of persistent fever. She was diagnosed with cervical pseudogout after closely examining the prolonged fever following *Staphylococcus epidermidis* bacteremia. Noninfectious diseases are frequent causes of unexplained fever in older adults, and systemic inflammatory diseases, such as cervical pseudogout, should be considered during examination.

## Introduction

Fevers in older adults have diverse causes, and the resultant fevers of unknown origin often complicate the diagnosis process. A fever of unknown cause (FUO) is a fever that exceeds 38.3 °C and persists for a minimum of three weeks. FUO can also be defined as a fever for which a precise diagnosis cannot be established after one week of hospitalization and close examination [1]. More than 200 causes of FUO have been reported, including infectious, inflammatory, and tumor-related diseases. Generally, history-taking and physical examination may reveal local signs and symptoms that can lead to a diagnosis [2]. However, this process is often complicated because multiple diseases modify the condition, especially in older adults [3]. There are no gold-standard tests for FUO diagnosis, and there is no unified opinion on which published algorithms and tests should be included in a comprehensive workup [4]. The first-line diagnostic approach for FUO includes general examination with various laboratory tests and basic imaging techniques, while the second line includes more advanced imaging techniques such as fluorodeoxyglucose positron emission tomography and tissue biopsy. More invasive measures, such as liver biopsy and exploratory laparotomy, are performed in sequence [5]. The aging of the population has led to a debate over the extent to which tests need to be performed. Therefore, it is necessary to consider appropriate methods of scrutiny in discussions with patients, families, and various professionals while avoiding a fall into ageism.

Diverse perspectives are needed to facilitate the scrutiny of fever in older adults. Older adults are affected by comorbidities, aging-induced physiologic changes, a decrease in homeostasis, and reduced activities of daily living and quality of life due to disease and aging [6]. So, among them, fever requires a closer examination for the causes of the FUO than the examinations conducted on younger patient populations. Infections are the most common cause in younger patients, while systemic diseases, such as temporal arteritis, polymyalgia rheumatica, malignancy, and drug fever, are common causes in older adults. An absence of fever may mask symptoms, and diverse comorbidities can complicate and confound the diagnosis [7]. In addition, communication difficulties primarily due to brain dysfunction and cognitive decline after stroke can impede access to appropriate medical care and increase the risk of avoidable adverse events by a factor of three [8].

Furthermore, diverse acute illnesses may lead to the development of new acute diseases, and in these cases, there may be multiple causes of fever. In this study, we describe a case of epidermal staphylococcal bacteremia of unknown cause that resulted in a fever and eventually led to the diagnosis of cervical pseudogout. Herein, we clarify the process of searching for and diagnosing the cause of FUO in an older patient and discuss effective methods of searching for the causes of FUO.

## Case presentation

A 94-year-old bedridden woman was brought to our hospital with a complaint of persistent fever. One month earlier, she also had a fever of unknown origin, and her family doctor performed a blood culture that was positive for *Staphylococcus epidermidis*. There were no other physical findings to suggest an apparent infection, and contamination of the sample was a possibility. Therefore, urinary tract infection and pneumonia were considered, minocycline hydrochloride was administered, and the fever resolved within one week. Thereafter, however, intermittent fever in the 38 °C range was observed at night for the previous ten days, and a wet cough was present with right pulmonary crackle. A blood culture performed by her family doctor again revealed staphylococci in the epidermis, and she was treated with minocycline hydrochloride and ciprofloxacin hydrochloride for one week. However, low-grade fever of 37.4 °C persisted with an inflammatory reaction. Her medical history included right cerebellar infarction, chronic heart failure, persistent atrial fibrillation, dementia, post gastrostomy syndrome, and gastric ulcer. Her medication history included edoxaban tosylate hydrate, furosemide, lansoprazole, tiapride hydrochloride, and trazodone hydrochloride.

During the visit to our hospital, her vital signs were as follows: temperature, 37.0 °C; blood pressure, 142/68 mmHg; pulse, 78 beats/minute (irregular), and SpO_2_, 95% (room air). A physical examination revealed no jugular venous distension or abnormal breath sounds; however, a systolic heart murmur radiating to the neck and extremities and pitting edema, mainly in the lower extremities, were observed. Blood tests showed inflammatory findings with high erythrocyte sedimentation rate and C-reactive protein (CRP), elevated brain natriuretic peptide, and hypoalbuminemia. Furthermore, urinalysis showed elevated erythrocytes and leukocytes (Table 1).

**Table 1:** The initial laboratory data.

Measure	Level	Reference range
White blood cells (/μl)	3.05 × 10^3^	3.5–9.1 × 10^3^
Neutrophils (%)	76.1	44.0–72.0
Lymphocytes (%)	13.1	18.0–59.0
Red blood cells (/μl)	3.05 × 10^6^	3.76–5.50 × 10^6^
Hemoglobin (g/dl)	10.0	11.3–15.2
Platelets (/μl)	2.32 × 10^5^	1.30–3.69 × 10^5^
Total protein (g/dl)	5.5	6.5–8.3
Albumin (g/dl)	2.4	3.8–5.3
Total bilirubin (mg/dl)	0.4	0.2–1.2
Aspartate aminotransferase (IU/L)	22	8–38
Alanine aminotransferase (IU/L)	11	4–43
Alkaline phosphatase (U/L)	85	106–322
Lactate dehydrogenase (U/L)	178	121–245
Blood urea nitrogen (mg/dl)	25.1	8–20
Creatinine (mg/dl)	0.74	0.40–1.10
eGFR (mL/min/L)	54.1	>60.0
Serum Na (meq/L)	137	135–150
Serum K (meq/L)	3.4	3.5–5.3
Serum Cl (meq/L)	100	98–110
Serum Ca (mg/dl)	8.3	8.8–10.2
Serum P (mg/dl)	4.1	2.7–4.6
Creatinine kinase (U/L)	25	56–244
C-reactive protein (mg/dl)	3.28	<0.30
Erythrocyte sedimentation rate (mm)	33	3–15
SARS-cov-2 antigen	Negative	
Urine testing		
Leukocyte	3+	-
Nitrite	-	-
Protein (mg/dl)	2+	-
Glucose	-	-
Urobilinogen	normal	
Bilirubin	-	-
Ketone	-	-
Blood	3+	-
pH	5.5	
Specific gravity	1.009	

**Figure 1:**
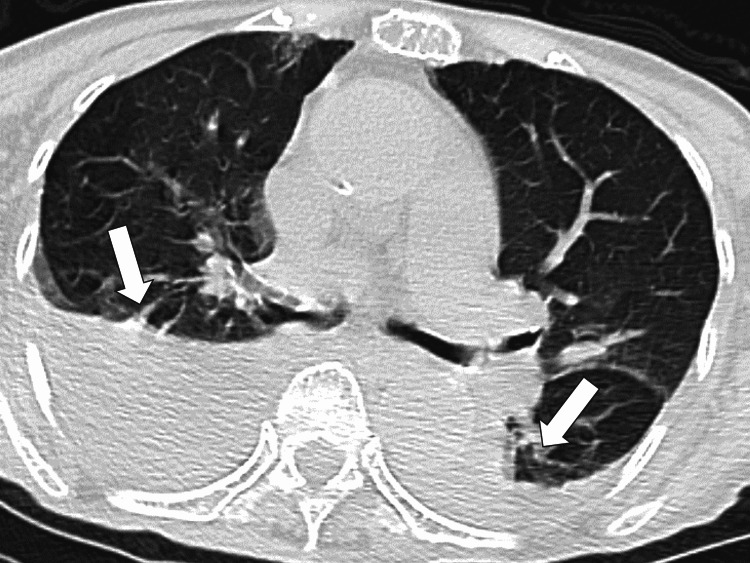
Initial computed tomography of the chest shows a bilateral pleural effusion.

A computed tomography (CT) chest scan showed increased pleural effusion predominantly on the right side and pitting edema in the lower extremities, with no significant change from one month earlier, suggesting no exacerbation of chronic heart failure (Figure 1).

A Gram stain of a urine sample showed numerous Gram-negative rods; however, blood cultures conducted after admission showed no bacteria.

**Figure 2:**
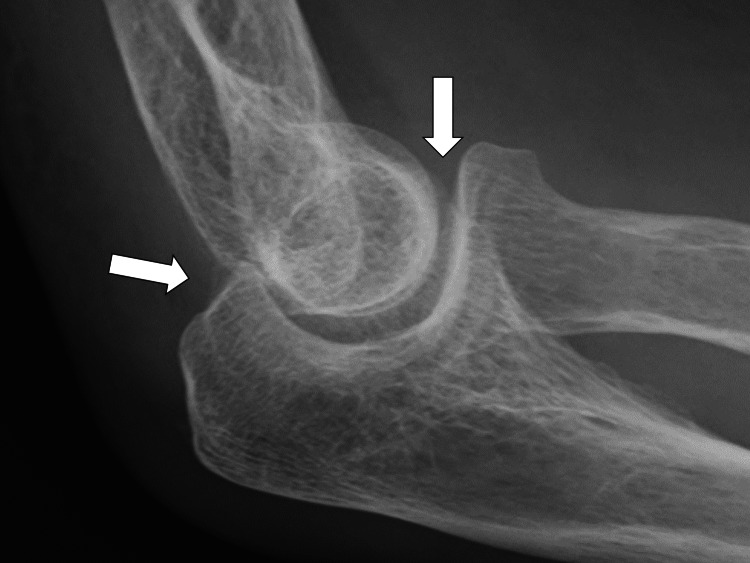
X-ray of the right elbow shows cartilage calcification.

Clinically, the patient was considered to have pyelonephritis and was treated with intravenous cefmetazole (4 g/day). A quick sequential organ failure assessment was not applicable, and there were no findings suggestive of sepsis. However, after admission, the patient continued to have a fever in the 37 °C range. A physical examination revealed neck pain and tenderness in both elbow joints bilaterally. Therefore, thorough cervical CT and radiographic examinations of the extremity joints were performed (Figure 2).

**Figure 3:**
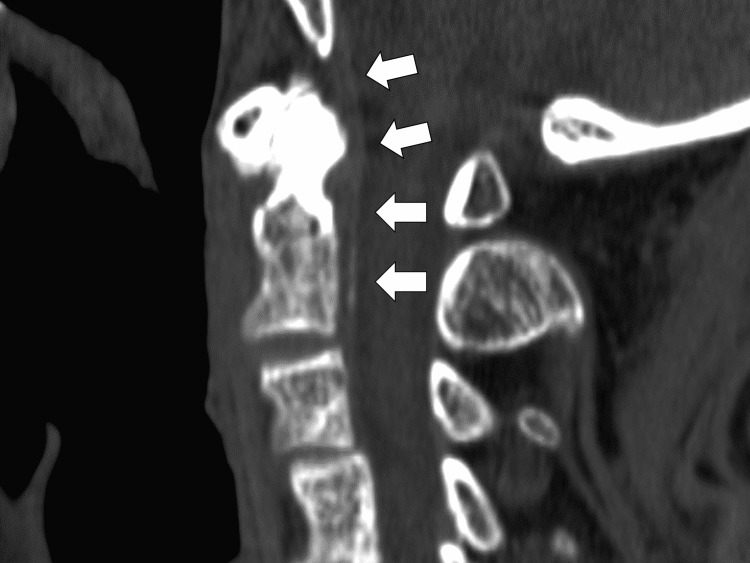
Computed tomography of the neck shows calcification of the dens and the posterior ligament of the cervical vertebrae.

The cervical CT showed hyperabsorption areas, suggesting calcification of the axial vertebral process and posterior longitudinal ligament. Accordingly, a diagnosis of pseudogout of the axial joint (crowned dens syndrome) was established (Figure 3).

Since the patient was older and had a history of gastric ulcers, treatment was started with 10 mg of prednisolone. The fever quickly resolved, and the patient's level of consciousness and appetite improved. The patient's progress was considered good, and she was discharged home nine days after the disease onset.

## Discussion

This case of cervical pseudogout was diagnosed after close examination of a prolonged fever following epidermal staphylococcal bacteremia. Noninfectious diseases are frequent causes of FUO in older adults, including systemic inflammatory diseases (rheumatic diseases, connective tissue disorders, vasculitis including temporal arteritis, polymyalgia rheumatica, and sarcoidosis). Therefore, it is necessary to scrutinize the patient for systemic conditions that do not usually cause fever in younger patient populations [9]. In this patient, infection was initially considered the cause of the fever since the patient had bacteremia before admission, and the fever was accompanied by an increase in the number of leukocytes in the urine at presentation. However, subsequent examination of the neck and joints revealed that the patient also had a noninfectious disease. Therefore, during the diagnosis of fever of unknown origin in older adults, the clinical course, physical examination, and other findings should be considered.

Pseudogout can occur in older patients with fever triggered by infectious disease, and, thus, close examination is necessary. In this case, a blood culture performed by the patient's family doctor was positive for *S. epidermidis*; however, no obvious source of infection could be identified. S. epidermidis is a commensal bacteria of human skin and is generally not pathogenic. Infections of non-immunocompromised patients and community-acquired infections do not commonly occur. However, given the patient's advanced age and multiple comorbidities, she was likely immunocompromised. In these cases, it is necessary to search for the source of infection rather than treat the patient while considering the culture sample to be contaminated. Some publications report the formation of superficial muscle abscesses due to *Staphylococcus aureus* bacteremia. In muscles near joints, there are also reports that deposition of calcium pyrophosphate crystals, as observed in pseudogout, may result in pseudo abscess formation and increased susceptibility to infection [10]. It should also be recognized that *S. aureus* is the primary causative organism of infective endocarditis and that a wide range of clinical manifestations and musculoskeletal complaints of infective endocarditis are relatively common, with reports of initial manifestation of musculoskeletal symptoms that may present as pseudogout-like symptoms [11]. Therefore, it is essential to consider that a significant disease, such as infective endocarditis, may underlie the differential diagnosis of arthralgia with bacteremia, even if the patient presents with pseudogout-like symptoms.

Cervical pseudogout may cause unexplained fever in older adults because fever usually occurs before the onset of cervical pain. A study of FUO in Japan reported that 21% of cases remained undiagnosed after a six-month follow-up of patients diagnosed with FUO [9]. However, recent advances in immunohistopathology and imaging have shown that the diagnosis of FUO is more straightforward [3,12,13], making the search for the cause of FUO more difficult. Crowned dens syndrome occurs in older adults (average age of 71.4 years) and is a rare clinical manifestation of calcium pyrophosphate deposition. It is caused by calcium pyrophosphate deposition around the C2 vertebrae and is usually characterized by neck pain (100%), neck stiffness (98%), fever (80.4%), and mostly elevated inflammatory markers on blood tests (88.3%) [14,15].

Determination by inflammatory response is also essential in FUO. In the present case, the patient had acute neck pain, fever, and elevated C-reactive protein. These findings are consistent with cervical pseudogout. In diverse clinical settings, patients with neck or occipital pain associated with neck stiffness and high inflammatory markers should be considered for Crowned Dens syndrome and evaluated with imaging [16]. Therefore, imaging evaluation was also performed in this case, and calcification of the axial vertebral dental process and posterior longitudinal ligament was observed. Cervical pseudogout is a rare condition that can be overlooked and is often diagnosed as a fever of unknown origin, which can, in turn, prompt the conduct of unnecessary tests to determine the source of the fever. In this case, early physical examination revealed cervical pseudogout as a differential diagnosis, and imaging evaluation led to the diagnosis of the condition, avoiding unnecessary invasive scrutiny.

In older patients with dementia, it is difficult to obtain a detailed history and establish an accurate diagnosis. In addition, older patients have multiple comorbidities that can complicate the diagnosis process [17]. In the present case, the patient's cognitive decline made it challenging to verbally communicate subjective symptoms [17,18]. Therefore, cervical pseudogout was diagnosed by examining physical findings related to noninfectious diseases, a frequent cause of unknown fever in older adults. In cases wherein the patient cannot provide accurate subjective symptoms for various reasons, the medical history may be obtained from the patient's cohabitant or caregiver, including general condition (level of consciousness, appetite, weight loss, and impact on daily activities), local symptoms (headache, respiratory symptoms, digestive symptoms, urinary tract symptoms, joint symptoms, and skin symptoms), and risk factors (medical history, travel history, history of illness with contact, and exposure to animals and insects) [17,18]. In addition, nurses and therapists who frequently intervene in daily care and during medical examinations may assist in history-taking to help notice new results at an early stage.

## Conclusions

In this case, an older patient with multiple comorbidities and compromised immune function was diagnosed and treated by continued close examination of laboratory and physical findings with suspicion of noninfectious disease, the most frequent cause of unknown fever in older adults. Even in cases wherein a noninfectious disease is suspected, it is still necessary to consider that severe disease-causing bacteremia may be present subclinically. Lastly, if the patient's subjective symptoms cannot be accurately identified because of cognitive decline, information sharing with nurses and therapists after admission and changes in the patient's condition from the perspective of the patient's roommates and caregivers may be helpful in the diagnosis of fever of unknown origin in older patients.
